# Physiology education in China: the current situation and changes over the past 3 decades

**DOI:** 10.1186/s12909-024-05395-1

**Published:** 2024-04-12

**Authors:** Xuhong Wei, Ting Xu, Ruixian Guo, Zhi Tan, Wenjun Xin

**Affiliations:** Department of Physiology, Zhongshan School of Medicine, Science and Technology Building, Sun Yat-Sen University, East Wing, 74 Zhongshan Road 2, Guangzhou, 510080 Guangdong People’s Republic of China

**Keywords:** Physiology, Medical education, Problem-based learning, Case-based learning, Integrated curriculum

## Abstract

**Objective:**

As an experimental biological science, physiology has been taught as an integral component of medical curricula for a long time in China. The teaching effectiveness of physiology courses will directly affect students' learning of other medical disciplines. The purpose of this study is to investigate the current situation and changes in physiology teaching over 30 years in Chinese medical schools.

**Methods:**

National survey was conducted online on the platform SoJump via WeChat and the web. The head of the physiology department in medical school was asked to indicate the information of physiology education from three periods: 1991–2000, 2001–2010, and 2011–2020. The responses of 80 leaders of the Department of Physiology from mainland Chinese medical schools were included in the study for analysis.

**Results:**

The survey showed that the class hours, both of theory and practice, had been decreased. During the past 20 years, the total number of physiology teachers, the number of physiology teachers who had been educated in medical schools, and the number of technicians had been reduced, whereas teachers with doctor’s degrees had been increased. In addition to traditional didactic teaching, new teaching approaches, including problem-based learning/case-based learning/team-based learning, integrated curriculum and formative evaluation systems, had been employed, mostly for more than 5 years, in some medical schools.

**Conclusion:**

The present study has provided historical data regarding the current status of physiology education in China and that in the past thirty years by showing that physiology education in China has developed quickly,even it faces many challenges.

**Supplementary Information:**

The online version contains supplementary material available at 10.1186/s12909-024-05395-1.

## Background

Physiology is an important foundational discipline in medical schools [1]. It studies how different cells, tissues and organs work together to maintain the normal function of the human body. The task of medical students is to learn how to diagnose and treat diseases. Therefore, it is necessary to first understand the functions of the normal human body by studying physiology, laying the foundation for subsequent courses, such as pharmacology, pathophysiology and clinical disciplines.

In 1998, the “Education Promotion Plan for the 21st Century” was published by the Ministry of Education of China. Since then, a massive increase in medical student enrollment has occurred [2]. Additionally, in 1998, stand-alone medical schools from the former Soviet model were merged into comprehensive universities in China to follow the model of medical education in the United States and other countries [2, 3]. These changes accordingly raised new challenges to medical education, for example, a rapid increase in the number of students without sufficient teachers and a lack of effective teaching strategies and methods. Thus, a survey on the current situation and changes in physiology education, including course hours and teaching staff, is necessary.

The traditional teaching model in physiology courses relies heavily on teacher-centered didactic lectures, with the students being given approximately 90 min of theoretical knowledge in the classroom in their second year of study. There were also a number of laboratory practices that ran concurrently with or subsequent to the lectures. Lecture-based learning (LBL) is good at transferring massive knowledge, the foundational cognitive skill from information professionals to students, but is often limited in facilitating the development of Bloom’s higher-order cognitive skills in students [4] due to passive acceptance of knowledge. Instructional strategies, such as problem-based learning (PBL), case-based learning (CBL) and team-based learning (TBL), which can promote active learning, have been widely adopted in medical education [5–10]. Their common merits involve developing cooperation among students, arousing consciousness of lifelong learning and improving problem-solving skills. In 2016, the "Chinese Undergraduate Medical Education Standards—Clinical Medicine Major" was released by the Ministry of Education, which aimed to develop student-centered and self-directed learning as the main content of educational strategies. This pointed out the direction for improving the level of medical education in China. Thereafter, an increasing number of student-centered learning methods, including PBL, CBL and TBL, are gradually being integrated into Chinese medical education. For example, problem-based self-designed experiments in physiology laboratory teaching are currently being adopted in Zhejiang University School of Medicine [11]. However, a national survey of the current PBL/CBL/TBL application status in physiology is still lacking.

In the 1950s, Case Western Reserve University first implemented an organ-system-based curriculum. In 1993, the curriculum reform of the Edinburgh World Medical Education Summit and the National Outstanding Doctor Training Plan opened the prelude to curriculum integration teaching in domestic medical colleges. In 1994, Reagan and Menninger reported on 10 years of experience by integrating physiology with other basic biomedical disciplines, such as anatomy, biochemistry, and pharmacology, in a PBL format [12]. In 2002, Shantou University Medical Schools first adopted an integrated curriculum in China [13]. In 2013, an integrated medical curriculum between basic medical courses and clinical curriculum was required in “several opinions of the ministry of health on implementing comprehensive reform of clinical medical education” [14]. Since then, the curriculum integration teaching model based on the concept of medical integrity and centered on the Organ system has become the new teaching reform. In 2020, the State Council General Office also stated “accelerating the innovative development of medical education and promote classroom reform in medical education by applying modern information technology in medical education” in China [15]. Under the new situation, reform in medical education has been accelerated, and the integration of modern information technologies in physiology teaching has been promoted.

In China, 11 broad categories, such as basic medicine, clinical medicine, stomatology, public health and preventive medicine, traditional Chinese medicine, were included in medical education. Clinical medicine is the main body of the medical education system in China, with 192 medical schools providing clinical medicine education [16, 17]. As a particularly important basic medicine, it is taught as a discipline-based curriculum that emphasizes one-sidedness but lacks the overall concept of medicine in most medical schools,. Integrated teaching can integrate physiology with other disciplines, such as anatomy, pharmacology or clinical curricula, in a unified manner, thereby strengthening students' cognition of disease from different dimensions and levels, which is beneficial for broadening students' vision and reducing repetitive and unnecessary teaching content. In the 1950s, an organ-system-based integrated curriculum was first implemented at Case Western Reserve University. The integrated curriculum of medical education in China began in the 1990s [18, 19]. In 2014, the "Deepening the cultivation of clinical medical talents through clinical practice and medical education collaboration" was issued by the Chinese Ministry of Education (MOE) [20]. This might greatly accelerate the reform of integrated medical courses.

A recent published study by Feng et al. has evaluated changes in Chinese medical schools for Physiology teaching over the last 20 years [21]. For the better development of physiology curricula, in the present study, we conducted a survey on physiology teaching in China to understand the current state and changes in the past 30 years, including course hours in theory and practice, faculty compositions, practice type and conducting time, and teaching approaches. Different to Feng’s study, in the present study we payed more attention to the changes of experiments type and the new teaching approaches. In addition to achieving the similar results as Feng’s study [21], we found that the total number of teachers in the physiology department had gradually decreased in the past 30 years, which was different from Feng’s study showing that the total number of physiology teachers was reported unchanged in most schools. We also found that the explorative and virtual experiments have developed quickly, which has not been reported previously. Moreover, our results also showed different integration content.

## Methods

### Study design

The main purpose of the study was to understand the current situation and changes in physiology education and teaching in the Chinese mainland, focusing on course hours, faculty compositions, practice type and conducting time, and teaching approaches. The changes in physiology teaching, particular the decline rate in course hours in the past 30 years, was the main outcome measures. Therefore, we had estimated the the decline rate by consulting literature in advance. A line of previous study has shown that a total of 83.33% of the surveyed schools have reduced their Histology and Embryology Education, which is also an important course in basic medicine in China [22]. We estimated the contact hour of physiology was reduced similarly. According to the formula Z^2^_1__-ɑ/2_*pq/d^2^, in which Z_1-ɑ/2_ = 1.96, *p* = 83.33%, q = 1–83.33% = 16.77%, d = 0.1*p = 8.33%, the estimated sample size was 79, which meant that we need to include decline rate in physiology course hours from at least 79 medical schools to achieve effectiveness. Accordingly, we conducted a nationwide survey of the top 100 medical schools (according to Evaluation Metrics (STEM) and 5- year total STEM [accumulative STEM (ASTEM)] http://top100.imicams.ac.cn/ASTEM/college), including different levels of ranked universities in China, including Project 985, Project 211 schools, and ordinary universities. All of them have had a five-year clinical medicine programs for at least ten years, as the present study investigated only five-year clinical medicine programs, which are the most popular medical program in China. Therefore, we thought the top 100 schools could represent the whole China and could meet the research needs. The present study was conducted on line from November 2020 to June 2021. Under the approval of the college research and ethics committee, a cross-sectional study was conducted among the directors of the physiology departments of the top 100 medical schools, excluding those Hong Kong, Taiwan and Macau, and these medical institutions that are distributed in various provinces. Traditional Chinese medical schools, specialist technology colleges, and medical schools without five-years medical programs were also excluded.

### Eligibility criteria for participants

Inclusion criteria for choosing participants for this study involved: (1) participants should be directors of the physiology departments, that usually had extensive practical experience in teaching and had experienced or witnessed changes in theoretical and laboratory teaching reform in physiology over the past 30 years. (2) participants should be from the top 100 medical schools, distributed throughout almost every provincial-level administrative division in mainland China. (3) The participants had completed their PhD or MD degrees and had taught physiology in medical schools for at least one year. (4) Both male and female could be included.

### Data collection

Quantitative data were generated from a self-administered survey questionnaire. The questions was first generated from the perspective of front line physiology teachers, who had extensive practical experience in teaching and had experienced or witnessed changes in theoretical and laboratory teaching reform in physiology over the past 30 years. Then the questionnaire was designed based on the published literature, discussed by the authors and tested by teachers from the corresponding author’s school and was subsequently revised based on the feedback to ensure clarity of the questions. Therefore, the description of each question was easy to understand and was structured elaborately, and more importantly, it is suitable to evaluate the teachers’ perspectives regarding the current situation and changes over the past 3 decades in physiology education in China. The questionnaire contained 26 main questions, 4 of which were jump questions. The questionnaire was designed based on the published literature (22). The survey was developed to collect factual information covering three main areas of physiology education: (1) the changes in course hours, including theory and practice, during the past 30 years. There were 11 questions. All the questions were filling in the blank except question 4. For example, question 2 was: What was the duration of physiological theory courses in clinical medicine at your university, from 2001 to 2010; (2) the changes in physiology teaching strategies and assessment. There were 15 questions. For example, question 12 was: The physiological experiment course in the clinical medicine major of your university is set as the following: (If the option includes exploratory experiments, please answer 12a. If the option does not include exploratory experiments, please choose no in 12a). There were 8 choices for question 12, including A. basic; B. comprehensive; C, explorative; D, basic and comprehensive; E, basic and explorative, F, comprehensive and explorative; G, basic, comprehensive and explorative practices; H, no. Question 12a was: What was topic selection method for exploratory experiments at your university? The choices for question 12 was: A, designed by students and tutored by teachers; B, designed by the students; C, designated by the teachers; D, No. (3) Changes in the teaching staff in physiology education. There were 10 questions. For example, question 19 was: What is the proportion of physiology teachers with doctoral degrees that are teaching clinical medicine currently at your university? The choices for question 19 was: A, ≤ 50%; B, 51%-70%; C, 71%-90%; D ≥ 90%. Hence there were a total of 37 questions in the whole questionnaire, including the last question requiring the participants to show their names and schools. The questionnaire is not a structured scale with similar scale anchors or values (The anchor ran from 1 = ‘Not at all’ to 5 = ‘To a very large extent’). There is weak correlation between questions and each question has different rating level. The directors of the physiology departments of these schools were in a messaging group in the WeChat application (Tencent Holdings Ltd., Shenzhen, China). The participants was first informed all about the study’s purpose, their right to withdraw at any time, and that their data would not be leaked. A two-dimensional code (SoJump, 2019, attached in Supplementary Material 1) invitation to participate in the online survey on the platform SoJump (Changsha Ranxing Information Technology Co Ltd., Changsha, China) was then sent to the WeChat group, a popular social media mobile application. Participation was voluntary and unrewarding. Respondents completed and submitted the questionnaire via mobile phone or computer, which has unique IP address, so that the authors could know whether one participants had submitted the answers twice with the same device. To increase their engagement and the authenticity of their answers, the participants were required to read the instructions before doing the survey. The contact information of the participants was also sought through personal contacts and websites. Most completed surveys were followed up with phone calls or email to confirm the accuracy of the information, to clarify obscure answers and to help the respondents complete omitted items if they were willing to do so. The results will not be adopted in statistics if the filling time was too short and the incomplete information could not be supplemented. To prevent the use of repeated responses from the same medical school, the respondents were required to show their institutions. The directors were also required to show their names to ensure that they submitted a single answer from each school. In addition, the answers could also give hints whether the participants had taken the survey seriously as some items from different questions confirm each other. For example, the number of participants that choose choice H in question 12, choice E in question 12a and choice G in question 13a are the same, which means the same content that the authors wanted to obtain was answered consistently by the participants, even the content was presented in different ways.

Finally, a total of 82 responses (from 51 female and 31 male participants) were finally identified as valid, however, 4 different participates from 2 schools were identified to have submitted the questionnaire simultaneously, and therefore only 80 medical schools have attended the survey. The surveyed schools were more than that in Xin Cheng’s study, which was 66 (22). Hence, the survey response rate was considered 80%. How the 82 directors from the 80 medical schools represent the overall total medical teachers are shown in Table 1.

**Table 1:** Distribution of the respondents in each province in China

School	Responser	Represents 2011–2020	Represents 2001–2010	Represents 1991–2000	School	Responser	Represents 2011–2020	Represents 2001–2010	Represents 1991–2000
1	1	**14**	**18**	**28**	41	1	**9**	**7**	**5**
2	1	**9**	**10**	**10**	42	1	**10**	**20**	**17**
3	1	**14**	**10**	**10**	43	1	**26**	**23**	**22**
4	1	**7**	**6**	**6**	44	1	**21**	**16**	**8**
5	1	**10**	**8**	**8**	45	1	**22**	**13**	**12**
6	1	**12**	**12**	**20**	46	1	**15**	**7**	**8**
7	1	**11**	**10**	**8**	47	1	**5**	**16**	**7**
8	1	**9**	**5**	**6**	48	1	**16**	**8**	**12**
9	1	**15**	**15**	**15**	49	1	**7**	**15**	**10**
10	2	**10**	**12**	**8**	50	2	**18**	**12**	**13**
11	1	**12**	**8**	**5**	51	1	**15**	**16**	**9**
12	1	**6**	**8**	**8**	52	1	**15**	**12**	**18**
13	1	**7**	**10**	**12**	53	1	**12**	**18**	**4**
14	1	**13**	**15**	**15**	54	1	**16**	**6**	**6**
15	1	**13**	**13**	**11**	55	1	**8**	**10**	**10**
16	1	**10**	**14**	**8**	56	1	**12**	**9**	**7**
17	1	**11**	**12**	**12**	57	1	**8**	**7**	**7**
18	1	**10**	**9**	**10**	58	1	**7**	**10**	**27**
19	1	**12**	**23**	**15**	59	1	**13**	**15**	**18**
20	1	**24**	**3**	**2**	60	1	**10**	**8**	**8**
21	1	**4**	**6**	**8**	61	1	**15**	**8**	**6**
22	1	**10**	**10**	**10**	62	1	**8**	**10**	**10**
23	1	**9**	**10**	**10**	63	1	**10**	**11**	**11**
24	1	**10**	**12**	**14**	64	1	**11**	**14**	**5**
25	1	**5**	**8**	**8**	65	1	**14**	**8**	**10**
26	1	**9**	**4**	**4**	66	1	**10**	**5**	**14**
27	1	**5**	**5**	**4**	67	2	**6**	**12**	**12**
28	1	**7**	**15**	**10**	68	1	**10**	**10**	**10**
29	1	**15**	**14**	**14**	69	1	**9**	**13**	**15**
30	1	**15**	**9**	**9**	70	1	**20**	**12**	**4**
31	1	**11**	**10**	**5**	71	1	**15**	**7**	**12**
32	1	**15**	**10**	**18**	72	1	**7**	**8**	**18**
33	1	**15**	**15**	**15**	73	1	**13**	**2**	**5**
34	1	**17**	**18**	**22**	74	1	**3**	**21**	**20**
35	1	**12**	**13**	**15**	75	1	**15**	**5**	**9**
36	1	**10**	**12**	**14**	76	1	**5**	**7**	**14**
37	1	**10**	**12**	**5**	77	1	**9**	**5**	**3**
38	1	**13**	**10**	**10**	78	1	**11**	**9**	**12**
39	1	**8**	**15**	**11**	79	1	**11**	**12**	**13**
40	1	**14**	**10**	**11**	80	1	**15**	**5**	**15**

### Statistical analysis

Statistical analysis questionnaires with missing items were considered ineffective and excluded from subsequent analysis. The data collected were tabulated in Microsoft Excel 2016. All statistical analyses were performed using GraphPad (Prism 8.0, San Diego, CA). One-way analysis of variance (ANOVA) (with the post hoc Tukey test) was performed to assess the physiological contact hours. For all tests, *P* < 0.05 was considered significant. The results are expressed as the means ± SD. Effect size was shown by Cohen’s d value, which is determined by calculating the mean difference between two groups and then dividing the result by the pooled SD, that is, Cohen’s d = (Mean2-Mean1)/SD pooled, where SDpooled = √(SD1^2^ + SD2^2^)∕2. To determine the internal consistency of the responses, Cronbach’s alpha test was used to analyze the data obtained from the questionnaires.

## Results

### The geographical distribution of the surveyed medical schools

Finally, the 80 medical schools that had attended the survey were distributed in 29 provinces/municipalities. The geographical distribution of the surveyed medical schools is summarized in detail in Table 2.

**Table 2:** How the 82 responses from the 80 medical scholls represent the overall total medical teachers is shown

	Number of schools					
	1	2	3	4	5	8
Province	Tianjin	Shanghai	Beijing	Henan	Hunan	Guangdong
	Hebei	Liaoning	Chongqing	Jiangsu	Jilin	
	Shandong	Shanxi (山西)	Hubei	Shanxi (陕西)	Heilongjiang	
	Qinghai	Sichuan	Jiangxi	Fujian		
	Yunnan	Inner mongolia	Anhui	Guangxi		
	Xizang		Guizhou			
	Xinjiang		Zhejiang			
	Hainan					

### Changes in course hours in the physiology curriculum

**Fig.1:**
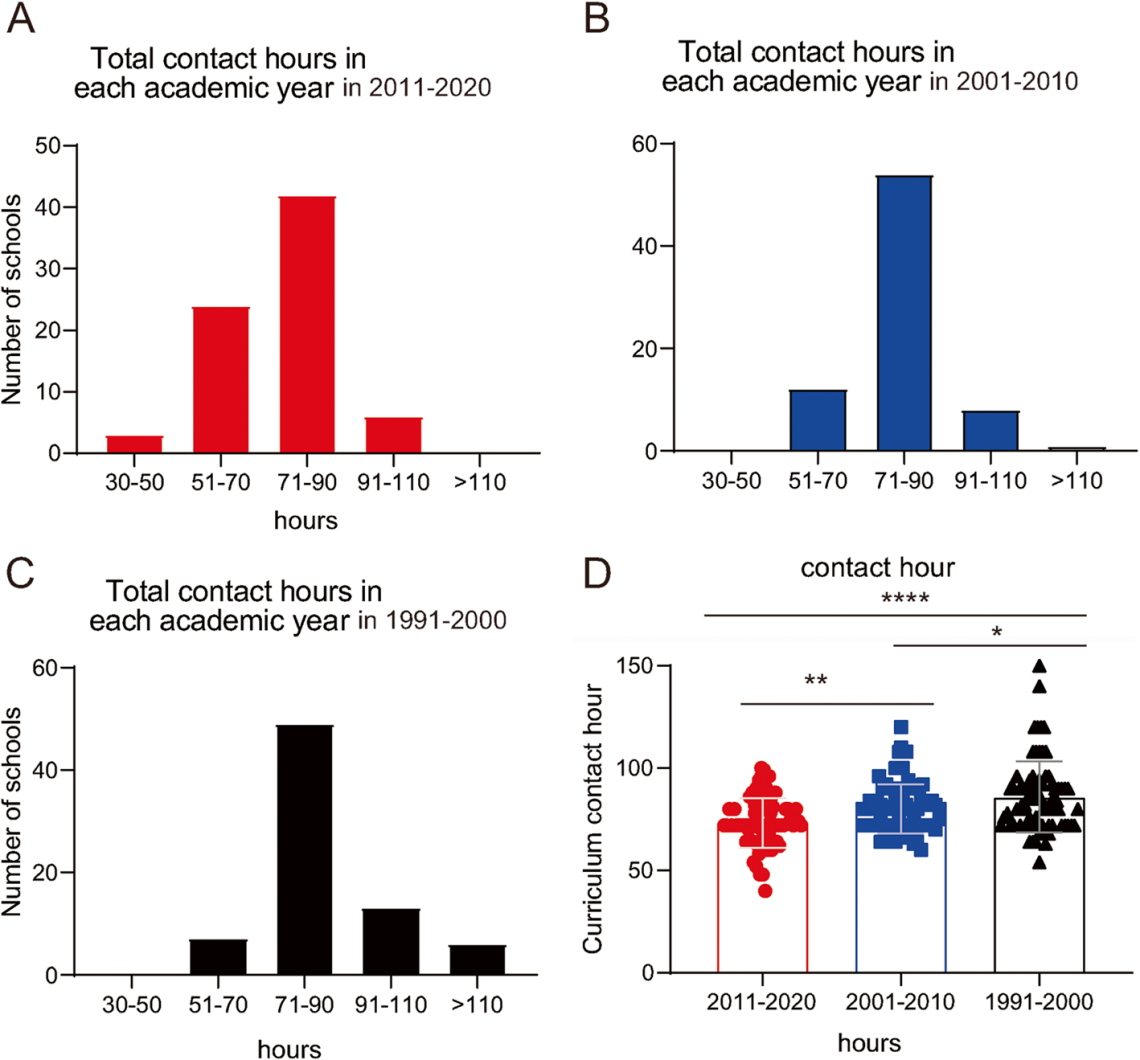
Survey of various aspects of changes of physiology curriculum in Chinese medical schools in the past 3 decades. **A**-**C** The bar charts show the numbers of schools with each range of the total number of contact hours of physiology in the 3 period as indicated in the surveyed Chinese medical schools. **D** Comparison of the average physiology (theory) contact hours in each academic year in the 3 periods. *P < 0.05; **P < 0.01, ****P < 0.0001 compared to the related group

This study focused on the current status and the changes in physiology education and teaching in China, however, some participants are not familar with early physiology teaching, making it challenging to get exact information. We have informed the participants that they could leave blank if they don't know the answer. At last we found that among the 80 medical schools, 76 participants supplied the exact number of their current physiology contact hours from 1991 to 2020. From Fig. 1, we can see that compared to 1991–2000, the schools with class hours > 110 had been gradually reduced in 2001–2010, and had disappeared in 2011–2020. In contrast, the schools with class hours in the range of 51–70 had been continuously increased in the past 3 decades (Fig. 1A-C). As shown in Fig. 1D, the average contact hours of physiology were gradually decreased in the past 3 decade. in the 76 medical schools (mean ± SEM, 73.2 ± 1.4 for 2011–2020, 80 ± 1.4 for 2001–2010, 85.9 ± 2 for 1991–2000, F(2,225) = 15.51, *P* < 0.0001).

### Changes in physiology teachers

**Fig.2:**
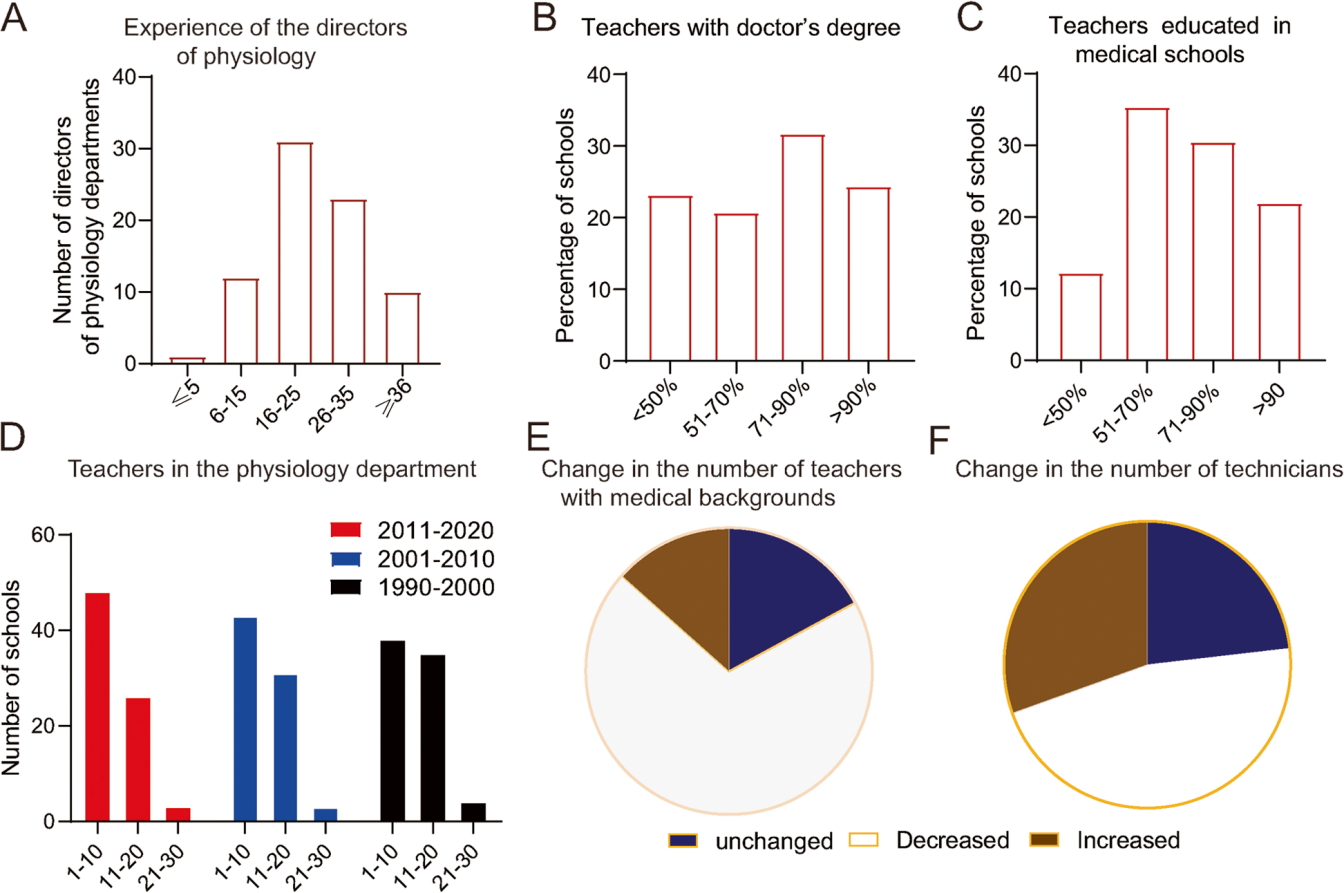
Survey of various aspects of physiology teachers at the surveyed Chinese medical schools. **A** The numbers of directors of physiology with each range of physiology teaching experience. **B-C**, each percentage range of physiology teachers with doctor’s degree (**B**) and with medical educational backgrounds (**C**). **D** The changes in the percentages of the total number of physiology teachers within the 3 periods. **E**, **F** Changes of the numbers of physiology teachers with medical educational backgrounds (**E**) and the number of technician staff (**F**) over the past two decades

The same survey was also conduced to understand the changes in physiology teachers, who are the primary resource for educational development. From the survey, it was found that most directors of physiology departments who responded to the survey were experienced in physiology teaching. As shown in Fig. 2A, among the 82 respondents, most of them have had a range of 26 to 35 years, even 10 had more than 36 years of teaching experience; only 1 had ≤ 5 years of experience. The present study also showed that in the majority of the medical schools, > 70% of the teachers had received their doctor’s degree (Fig. 2B), demonstrating that the physiology teachers had good educational backgrounds. Furthermore, in 10, 29, 24 and 17 of the surveyed 80 medical schools, ≤ 50%, 51%-70%, 71%-90%, and ≥ 90% of the teachers had been educated in medical schools, respectively (Fig. 2C). A massive increase in student enrollment in medical schools has occurred since 1998 in China. To understand whether there was a sufficient number of physiology teaching staff to ensure teaching quality in China, the appropriate number of teachers who had worked at the same time in the physiology department in the past 30 years was quantified. The results show the numbers of surveyed medical schools with different teacher numbers in the three periods of 1991–2000, 2001–2010, and 2011–2020. The results showed that the number of schools that had teachers ranging from 1–10 increased continuously, whereas the number of schools that had teachers ranging from 11–20 decreased continuously in the pat 3 decades (Fig. 2D). Compared to 20 years ago, most of the surveyed schools had decreased numbers of teachers possessing medical doctor’s degrees in the physiology departments (Fig. 2E).

Technicians contribute greatly to physiology education by preparing the material and maintaining experimental instruments and related software. The survey showed, however, that the number of technicians, compared to that 20 years ago, decreased in 46.3% of Chinese medical schools and increased in only 30.5% of medical schools (Fig. 2F), suggesting that there have not been enough technicians in recent years.

### Changes in physiology practice

**Fig.3:**
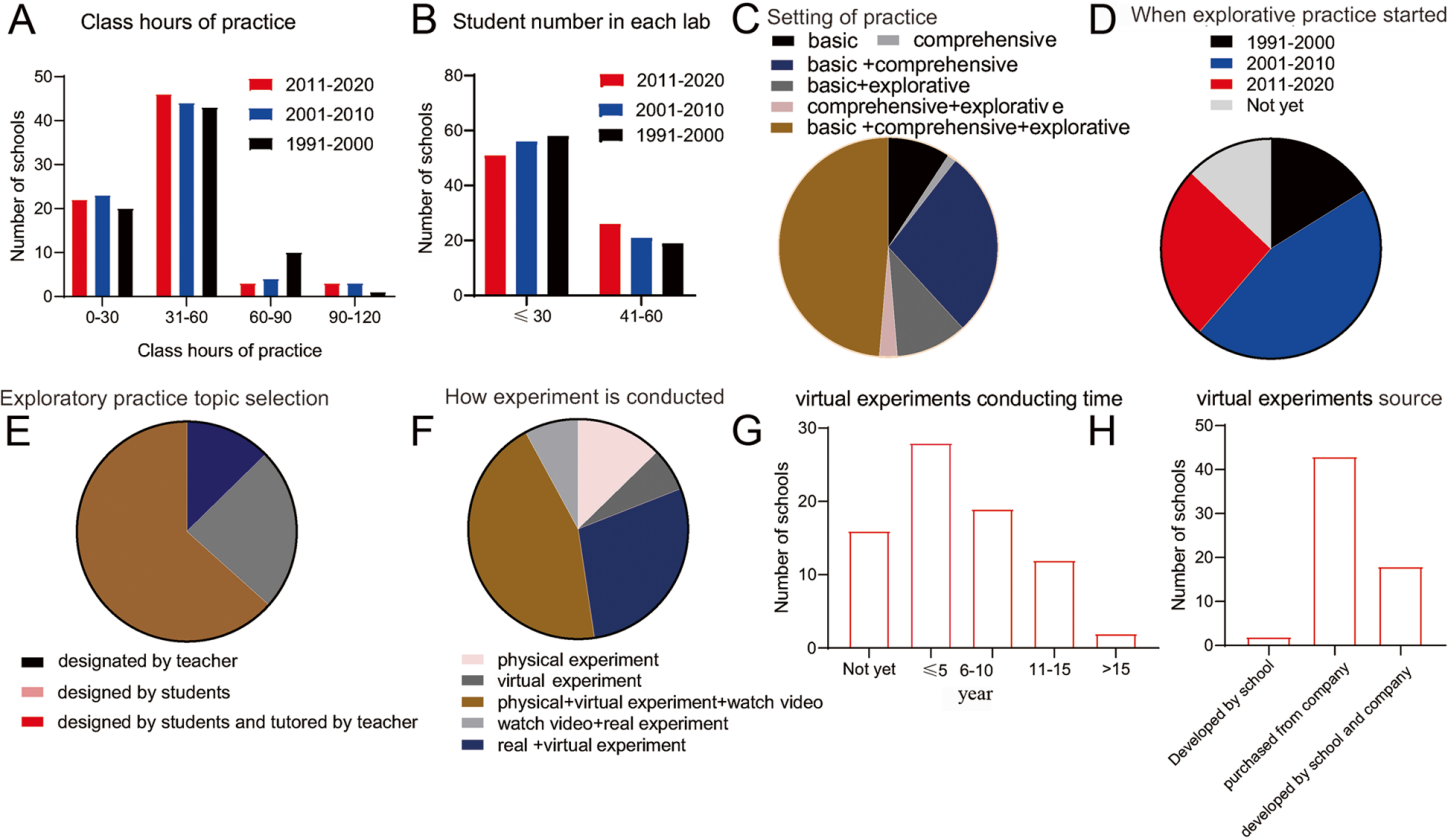
Survey of various aspects of physiology laboratory practice in Chinese medical schools.**A-B** The bar charts show the numbers of schools with each ratio of practice courses hours for physiology (**A**), with each range of the number of students in each laboratory (**B**).**C**, the setting types of practice in the surveyed Chinese medical schools are shown. **D**, **E** The pie charts show the percentage of schools that started explorative practice in the 3 period (**D**) or how the explorative practice topics was selected (**E**) compared to those 20 years ago in the surveyed Chinese medical schools. **F** The pie chart show the percentage of schools that employed physical experiments, virtual experiments, physical experiments combined with virtual experiments, physical experiments combined with watching videos, physical experiments combined with virtual experiments and watching videos, respectively. **G **The bar charts show the numbers of schools with each range of virtual experiments conducting time. **H **The bar charts show the numbers of schools with each virtual experiment sources

Experimentation is fundamental to scientific methods of physiology. A range of 31 to 60 course hours in physiology practice in clinical medicine major in China was predominant. The course hours in the range of 60–90 greatly decreased during the period of 2000–2009 compared to 1990–1999 (Fig. 3A). The numbers of schools that having less than 30 students in each laboratory had decreased continuously from 1991 to 2000, whereas the numbers of schools that having 41–60 students in each laboratory were gradually increased since 1991 (Fig. 3B). In each laboratory in the medical schools in China, usually only one teacher tutors all the students when they are doing the practice. Therefore, an increased number of students in each laboratory will lessen the amount of time that the teacher can communicate with each student. According to the survey results, regarding the types of practice, basic, comprehensive, basic and comprehensive, basic and explorative, comprehensive and explorative, and basic, comprehensive and explorative practices were conducted in 9%, 1.3%, 29.5%, 10.3%, 2.6%, and 47.4% of the surveyed schools, respectively (Fig. 3C).

Regarding explorative practices, 28 schools had adopted them for less than 5 years, 19 for more than 5 years but less than 10 years, 12 for 11–15 years, and only 2 for more than 15 years. Sixteen schools had not yet adopted explorative practices (Fig. 3D).

In 63.4% of the surveyed schools, the exploratory practice topic was designed by students and tutored by teachers. In 23.9% and 12.7% of the surveyed schools, it was designed by the students or by the teachers, respectively (Fig. 3E).

It is difficult to control testing variables in physical experiments. Videos that showing experiments procedures and virtual experiments are becoming considerable options that have greatly altered physiology teaching. A total of 10.3%, 6.4%, 24.4%, 35.9% and 23.1% of the surveyed schools employed physical experiments, virtual experiments, physical experiments combined with virtual experiments, physical experiments combined with watching videos, physical experiments combined with virtual experiments and watching videos, respectively (Fig. 3F). Virtual experiments had been employed for less than 5 years in 28 and 19 of the surveyed schools, whereas it had been employed for more than 10 years in only 14 schools. In 16 schools, virtual experiments have not yet been employed (Fig. 3G). Furthermore, the virtual experiment sources were purchased from the company in most of the surveyed schools, secondly developed by the school and the company together. Only 2 schools developed the virtual experiments by themselves (Fig. 3H).

### Changes in physiology teaching approaches

Medical education in the West has undergone several influential reforms, such as the development of PBL at McMaster University in the 1960s [23] and an integrated curriculum at Newcastle University and Case Western Reserve University in the 1990s [18, 19]. The survey was conducted to understand whether these reforms have also influenced physiology education. The results showed that in addition to traditional didactic teaching, teaching methods have also been innovated in some medical schools in China.

**Fig.4:**
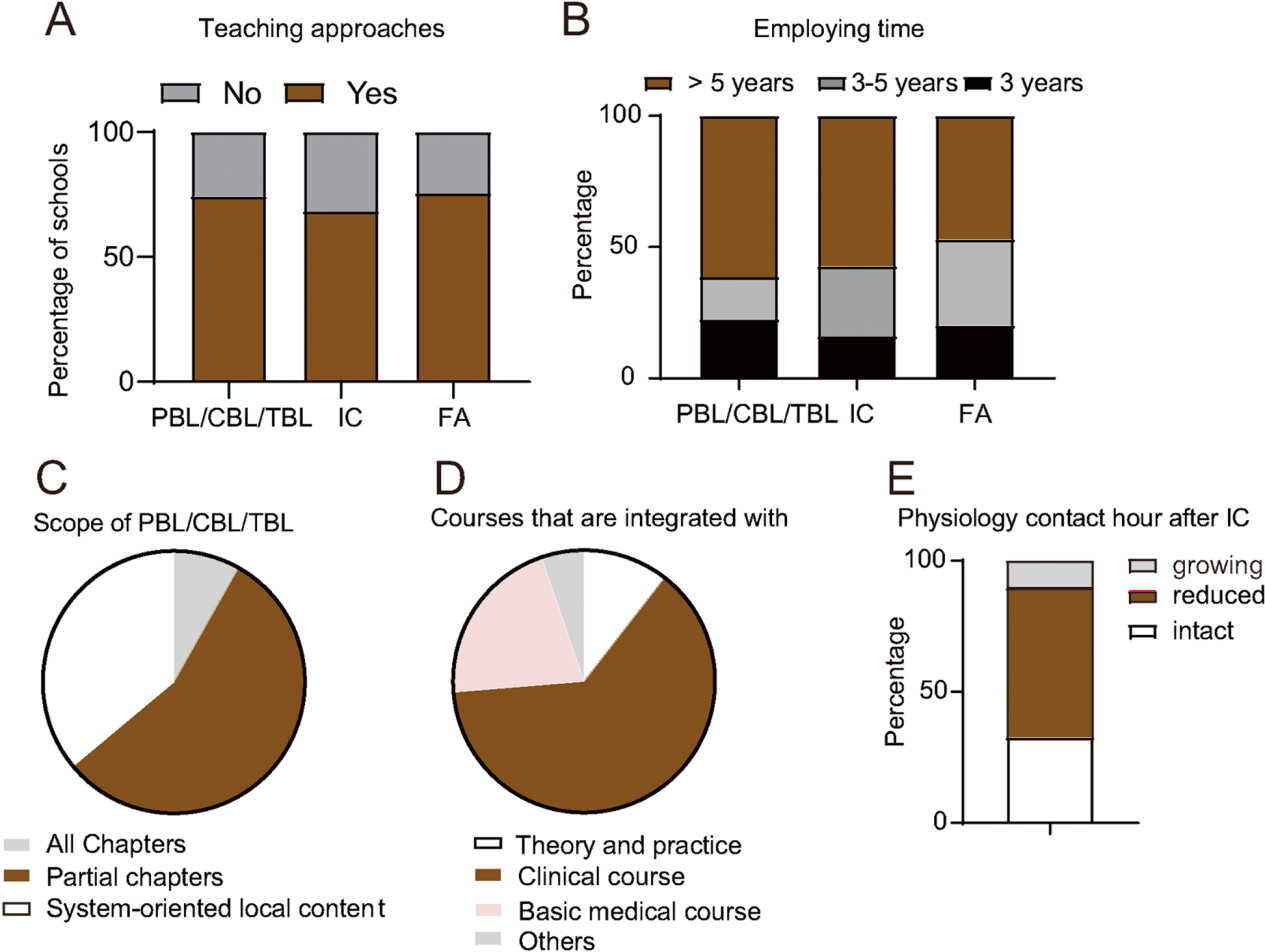
Survey of various aspects of physiology teaching strategies and assessments in Chinese medical schools. **A** The bar charts show the percentages of the medical schools that have or have not implemented PBL/CBL/TBL, integrated curricula, and formative assessments. **B **The percentages of the medical schools with each range of employing time of PBL, integrated curricula, and formative assessments. **C** The percentages of the medical schools that have employed PBL/CBL/TBL in all chapters, partial chapters or system-oriented local content of physiology. **D** The pie charts show the percentages regarding with which course physiology have been integrated with. **E**, How the contact hour of physiology changed after integration

At the time survey, PBL, CBL or TBL had been implemented in 74.4% schools, integrated curriculum models had been tried in 68.2% medical school, and formative evaluation systems had been established in 75.1% schools (Fig. 4A). In the schools that had tried PBL, CBL or TBL, 61.3% of them had experience of more than 5 years). 57.1% of the schools that had implemented integrated curriculum models for more than 5 years. In addition, 46.9% of the schools had tried formative evaluation systems for more than 5 years (Fig. 4B).

Moreover, the survey also demonstrated that over half of the schools (55.7%) implemented the PBL/CBL/TBL curriculum in partial chapters of physiology textbooks. A total of 36.1% had implemented system-oriented local content, and only 8.1% had implemented it in all chapters of physiology textbooks (Fig. 4C). A total of 63.1% of the schools that reported implementing integrated curricula also reported integration with clinical sciences, 21.1% with basic medical science, 10.5% reported integration theory with practice, and 5.3% reported integration with other curricula. At the time of the survey, curricular integration between theory and practice was reported in 10.5% of the surveyed schools. Integration with clinical sciences and other basic medical sciences was reported in 63.2% and 21.1% of the surveyed medical schools, respectively (Fig. 4D). Furthermore, at least half of the schools that had conducted integrated curricula reported reduced contact hours in physiology. A total of 32.8% and 10.3% reported intact and growing contact hours after integration, respectively (Fig. 4E).

## Discussion

Physiology education is a microcosm of the reform and development of the medical education in the Chinese mainland. Hoping to improve the quality of preclinical medical education, the present study was undertaken to present an overview of current status and the changes in physiology education, focusing on course hours, teaching strategies and student assessments, teaching staff in China by conducting a nationwide survey.

A total of 82 responses were finally included in the reports, representing 80 top medical schools. The survey focused on the teaching of clinical medicine students, which usually comprise the largest programs at medical schools. The respondents covered most of the top 100 Chinese medical universities/schools; therefore, the information collected by the survey could represent Chinese medical universities/schools. The results showed that the number of teaching hours spent on physiology at medical schools in China has been significantly reduced, in the past 30 years. In addition, both the quantity and composition of teachers have changed considerably. Traditional didactic teaching is still predominant even though new teaching approaches, including problem-based learning/case-based learning/task-based learning, integrated curriculum and formative evaluation systems have been conducted.

It is well known that small group teaching and exposure to practicals benefit learning, however, the survey showed that both the lecture contact time and laboratory practice hours of physiology in each academic year had decreased in the past 30 decades. Decreased course hours on physiology have occurred worldwide not only in recent times but also in earlier times, both in China and overseas. It has been reported that from 1955–56 to 1985–86, laboratory hours devoted to animal and human physiology declined by 92% [24]. Consistently, the recent study by Feng et al. also shows that the physiology class hours and the ratio of physiological theory to laboratory have been decreased over the last 20 years [21]. The reason that physiology class hours are decreased, however, is complicated. At present, an increasing number of students are using internet-based e-learning, such as watching videos. Thus, one important reason for decreased physiology hours is the construction and application of online open courses, which can enable students to learn everywhere at any time by removing temporal-spatial barriers. One other reason for reduced course hours is perhaps to save time for students to do scientific research and for clinic curriculum, which is catering to the demands of modern medical education. The third reason might also be the outcome of educational advancement, that is, the students had been taught some of the physiology knowledge at high schools or even middle schools and there is no need to repeat teaching these knowledge in universities.

Undoubtedly, a high ratio of qualified teachers to students is desirable for medical education. Unfortunately, the survey showed that the total number of teachers in the physiology department had gradually decreased in the past 30 years, in contrast to the rapidly expanding student enrollment [25]. The recent study by Feng [21], however, has shown that the total number of physiology teachers remains unchanged rather than decreased. The reason for the difference, however, is still not clear. In the present study, we surveyed the directors of physiology discipline, whereas Feng’s study surveyed the heads or senior teachers. The different survey subjects might affect the survey results.

For the teaching experience of the respondents, the data showed that 98.8% of the respondents had over 5 years of teaching experience and most of them have had a range of 26 to 35 years, even 10 had more than 36 years of teaching experience, demonstrating that the directors have rich experience. There is no doubt that rich teaching experience is good to education. From another perspective, however, this data also suggest that the directors have a relatively old age, and perhaps it is getting difficult for them to accept new teaching strategies. Moreover, consistent with Feng’s study [21], our results showed that teachers possessing doctor’s degrees has increased, whereas teachers with medical education backgrounds has decreased. There are perhaps two reasons that contribute to these phenomena. First, student enrollment has been greatly expanded in most Chinese medical schools, and it is becoming increasingly difficult for graduates to obtain appropriate jobs. To alleviate employment pressure and improve competitiveness, they must pursue a PhD career. Second, fewer students with medical education backgrounds are willing to pursue a career in the full-time teaching of basic medical sciences [2], possibly because the income gap has widened further. Third, universities are excessively emphasizing scientific research achievements when recruiting the teaching staff and having a doctoral degree is the most basic requirement for entering the university. The increasing number of teachers with PhD degrees has two sides to physiology teaching. On one side, it helps to cultivate students' scientific research thinking as the teachers have received well training in doing scientific research. On the other side, teachers are often overburdened by spending too much time doing their own scientific research, and therefore, the attention that can be paid to teaching undergraduates will be greatly decreased. Some teachers would even think that teaching is a waste of time, so that the universities have to consider that attending undergraduate courses for a certain amount of time as one of the basic evaluation indicators. The decreasing number of teachers with medical education backgrounds suggest that the physiology teachers have difficulties in connecting with clinical practice during teaching. To ensure the teaching quality, on the one hand, medical schools should help the physiology teachers to cultivate the transforming medical concepts by establishing combined teaching teams between basic and clinic. On the other hand, medical schools should take strategies to reduce the pressure of the teachers from scientific research, so that they are willing to spend more time in teaching.

Every understanding or conclusion of physiology is obtained from practice. Practice also assists students in applying physiology to clinical applications. Regarding explorative practices, 28 schools had adopted them for less than 5 years, 19 for more than 5 years but less than 10 years, 12 for 11–15 years, and only 2 for more than 15 years. 16 schools have not yet adopted explorative practices (Fig. 4d).

Experimental courses are a very important part of physiology teaching. By conducting experimental courses, students' observation abilities and hands-on abilities can be better cultivated. However, some physiological experiments are time consuming or require expensive equipment. Furthermore, testing variables in physical experiments is difficult. Experiments on live animals also require high levels of biological security. The application of virtual experiments can effectively solve these difficulties by enabling students to experience the experimental process. The survey showed that virtual experiments are adopted by more and more schools, though most schools chose to purchase the virtual experiment sources by the companies. This results indicate that teaching strategies have been greatly impacted by the development of artificial intelligence. A recent study has introduced how to build an electronic standardized patient (ESP) based-virtual human body system powered by the real-time human physiological parameters in the teaching of human physiology. These ESP-based virtual simulation projects presumably becomes a considerable option for the first-class course construction in physiology [26]. In another study, the effectiveness of virtual labs in practicing biochemical experiments was assessed and the student's feedback regarding this tool was examined, showing that using virtual laboratories is effective in delivering practical parts of basic medical experiments to medical students and that the students have positive attitude toward using virtual laboratories in the practical sessions of a Medical Biochemistry course [27]. The authors believe that in the future, an increasing number of medical schools will employ combined virtual experiments and traditional experiments, as they can complement each other. Integrating medicine and industry will promote the progress of the medical education.

The high enrollments also raise the question that more students learn in the same class and share one teacher. With reduced teachers and lecture contact time hours, it is thus a challenge for departments to adequately organize teachers to maximize student learning and allow for student-centered teaching approaches. To compensate for the consequences of insufficient offline teaching, online teaching platforms that help to integrate and utilize teaching resources should be vigorously advanced. Currently, MOOCs education platforms such as the Chinese University MOOCs platform, XuetangX online platform, Zhihuishu MOOCs platform, and Chinese MOOC platform have been vigorously developed [21]. Microlectures are also popular in China. These online learning platforms are of great significance in cultivating students' professional knowledge and enhancing their innovative abilities. However, there are still issues that need to be improved, for example, teachers' online teaching ability needs to be improved, and students' autonomous interest in online learning needs to be stimulated.

For a long time, medical education, requires the medical students first learn basic and biomedical sciences and then move to clinical sciences. It emphasizes one-sideness but lacks the overall concept of medicine, has posed new challenges to the current medical training model and curriculum systems. In addition, the patients are presented in a totally different way as the traditional medical education. Integrated teaching can integrate different disciplines in a unified manner, thereby strengthening students' cognition of disease from different dimensions and levels, which is beneficial for broadening students' vision and reducing repetitive and unnecessary teaching content. It has been proven that the integrated teaching concept centered on organ systems benefits students’ early formation of medical concepts [28, 29]. At the time of survey, the majority of the medical schools employed integrated curriculum models. At the time of the survey, integration between physiology and clinical sciences (vertical integration) was reported in more than half of the schools. Integration physiology between other basic medical sciences (horizontal integration) was employed fewer than vertical integration, whereas integration between theoretical lectures and practice sessions was least adopted. These results are consistent with a previous study showing that most curricula for medical education have been integrated horizontally and vertically. Most of the integration was integrated vertically between basic and clinical sciences, yet an vertical integration with humanism, and health population in the vertical axis, not only in the early years but also throughout the curriculum is also needed [30]. Furthermore, most of the schools with integration courses had decreased contact hours in physiology. These results are consistent with previous reports that in a vertically integrated curriculum the time spent on classroom education gradually reduces across the years, while the time on clinical practice increases [31]. These results also indicate that medical educators have realized that the old curriculum system is not conducive to cultivate systematic clinical thinking patterns, and have already taken steps towards reforms. Reduced classroom hours but enhanced graduation requirement indicated the teaching strategies must be improved. Furthermore, the teaching quality evaluation systems must also be improved. Problem-based learning is an approach that is often used with the aim of creating curricular integration. To improve teaching strategies, PBL, CBL or TBL was implemented in most of the surveyed schools, with more than 5 years of experience.In addition, 46.9% of the schools had tried formative evaluation systems for more than 5 years.

### Limitation of this study

Not all the medical schools that provide five-year clinical medicine programs were included in the study, as the sample was not selected randomly.

## Conclusions

The present study has provided historical data regarding the current status of physiology education in China and that in the past thirty years. Physiology is still mainly taught as a discipline-based curriculum in most medical schools, even though it is integrated with other disciplines. Physiology education in China faces many challenges, such as decreased course hours and decreased teachers with medical backgrounds. Although innovative teaching strategies have been employed in some medical schools, traditional didactic methods are still mostly used. Overall, the present study helps to understand the current status of physiology education in China and raises some concern for the better development of physiology education. Although the sample may not be truly representative of whole China, they were representative of the top 100 medical schools in the mainland of China.

### Supplementary Information

**Supplementary Material 1.**

## Data Availability

Data and materials can be obtained from the corresponding author upon request.

## Abbreviations

LBL: Lecture-based learning
PBL: Problem-based learning
CBL: Case-based learning
TBL: Team-based learning
MOE: The Chinese Ministry of Education
STEM: Science and technology evaluation metrics
