# Supplementary material for: Physiology education in China: the current situation and changes over the past 3 decades
Source: BMC Med Educ. 2024 Apr 12;24:408. doi: 10.1186/s12909-024-05395-1 (PMC11015638; doi:10.1186/s12909-024-05395-1)
Supplement: Supplementary file 1 — Supplementary Material 1. [file 12909_2024_5395_MOESM1_ESM.docx]

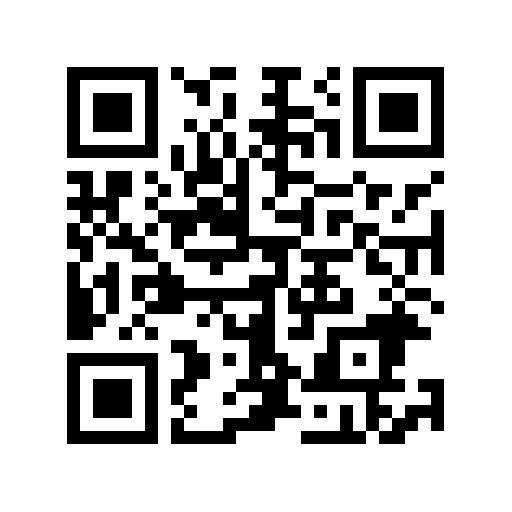


Supplementary Material 1

A two-dimensional code invitation to participate in the online survey on the platform SoJump.
